# Architecture and prototypical implementation of a semantic querying system for big Earth observation image bases

**DOI:** 10.1080/22797254.2017.1357432

**Published:** 2017-08-11

**Authors:** Dirk Tiede, Andrea Baraldi, Martin Sudmanns, Mariana Belgiu, Stefan Lang

**Affiliations:** ^a^ Department of Geoinformatics – Z_GIS, University of Salzburg, Salzburg, Austria; ^b^ Department of Agricultural and Food Sciences, University of Naples Federico II, Portici, Italy

**Keywords:** Big data, Earth observation, Level 2 product, spatiotemporal objects and events, array database, semantic content-based image retrieval

## Abstract

Spatiotemporal analytics of multi-source Earth observation (EO) big data is a pre-condition for semantic content-based image retrieval (SCBIR). As a proof of concept, an innovative EO semantic querying (EO-SQ) subsystem was designed and prototypically implemented in series with an EO image understanding (EO-IU) subsystem. The EO-IU subsystem is automatically generating ESA Level 2 products (scene classification map, up to basic land cover units) from optical satellite data. The EO-SQ subsystem comprises a graphical user interface (GUI) and an array database embedded in a client server model. In the array database, all EO images are stored as a space-time data cube together with their Level 2 products generated by the EO-IU subsystem. The GUI allows users to (a) develop a conceptual world model based on a graphically supported query pipeline as a combination of spatial and temporal operators and/or standard algorithms and (b) create, save and share within the client-server architecture complex semantic queries/decision rules, suitable for SCBIR and/or spatiotemporal EO image analytics, consistent with the conceptual world model.

## Introduction

### Semantic content-based image querying

Vision (image understanding) is a cognitive process responsible for scene-from-image representation, ranging from local syntax of individual objects to global gist and spatial layout of objects in the 4D spatiotemporal scene domain, including multiple plausible semantic scene interpretations and even emotions (Rodrigues & du Buf, 2007). Vision is inherently ill-posed, affected by a 4D to 2D data dimensionality reduction problem, responsible for occlusion phenomena, and by a semantic information gap, from quantitative sub-symbolic ever-varying sensations, specifically, 2D sensory data in the image domain, to stable symbolic percepts in the 4D spatiotemporal scene domain (Matsuyama & Hwang, 1990).

Traditional content-based image retrieval (CBIR) systems, including photographic image search engines such as the Google Image Search, support human–machine interaction through queries by metadata text information, by image-wide summary statistics or by either image, image–object or multi-object examples (Gudivada & Raghavan, 1995; Seidel, Schroder, Rehrauer, Schwarz, & Datcu, 1998; Shyu et al., 2007; Smeulders, Worring, Santini, Gupta, & Jain, 2000). Queries by image examples typically compute statistical similarities between numeric sub-symbolic low-level vision variables (e.g. pixel values, texture parameters) extracted from a reference and a test image pair without extracting high-level vision semantics for scene-from-image reconstruction. New approaches (e.g. used in Google+ Photo Search) are applying deep convolutional neuronal networks based on millions of training data to categorize and label photographs (Krizhevsky, Sutskever, & Hinton, 2012; Long, Shelhamer, & Darrell, 2014).

In the remote sensing (RS) domain, Earth observation (EO) CBIR systems support human–machine interaction through queries by either metadata text information or image-wide summary statistics (Shyu et al., 2007). For example, the popular US Geological Survey (USGS) Earth Explorer and the European Space Agency (ESA) Sentinel Scientific Data Hub allow a user to choose a geographic area of interest (AOI), a target timespan and some textual metadata, such as the name of the mission, image path/row, data category. The sole EO image filtering criterion they support is input with a maximum cloud-cover percentage value to be user-defined. This scalar threshold is compared with a cloud-cover quality index estimated offline for every EO image stored in the database. Any image-wide quality index (summary statistic) neither refers to the user-selected AOI nor provides information about its geospatial distribution across the AOI.

Unlike a traditional CBIR system, a semantic CBIR (SCBIR) system is expected to cope with spatiotemporal semantic queries such as “retrieve all images in the database where a lake is not covered by clouds and larger than a certain area”. Such an SCBIR system must rely on image understanding as a pre-condition for SCBIR. This makes the SCBIR problem at least as difficult (or ill-posed) as vision. Since computer vision is still an open problem, this may explain why very few SCBIR system prototypes have been presented in the RS and computer vision literature (Dumitru, Cui, Schwarz, & Datcu, 2015; Li & Bretschneider, 2004). To our best knowledge no SCBIR system in operating mode is available to date. We define an information processing system in operating mode if it scores “high” in a minimally dependent and maximally informative (mDMI) set of outcome and process quality indicators (QIs), encompassing accuracy, efficiency, degree of automation, scalability, robustness to changes in input data as well as to changes in input parameters, timeliness from data acquisition to product generation and costs in manpower and computer power (Baraldi & Boschetti, 2012).

In the RS domain, the EO SCBIR system prototype proposed by Li and Bretschneider (2004) adopts, first, a relational database to store planar information layers (symbolic strata) in a scene classification map (SCM) whose legend (codebook) is a dictionary of target land cover (LC) classes (codewords). Second, it employs a semi-automatic pixel-based statistical classifier. Because colour information is the sole visual feature available at pixel level, any pixel-based classifier ignores spatial information in the image domain. This is in contrast with the fact that, since chromatic and achromatic biological vision systems are nearly as effective in scene-from-image representation, spatial information dominates colour information in both the 2D image domain and the 4D spatiotemporal scene domain (Matsuyama & Hwang, 1990). Another EO SCBIR prototype, called Earth Observation Image Librarian (EOLib), was recently proposed by Dumitru et al. (2015). EOLib is built upon a support vector machine (SVM) for 1D image classification, where the 1D vector data sequence consists of image convolutional values generated by 2D spatial filters. Any inductive learning-from-data algorithm, such as an SVM, requires a priori knowledge in addition to data to become better conditioned for numerical solution (Cherkassky & Mulier, 1998). As a consequence, EOLib can be considered as semi-automatic only. In addition, any 1D image classifier is an orderless encoder invariant to permutations, where spatial topological information in the (2D) image domain is lost.

Our conjecture is that existing EO CBIR systems support no semantic querying because they lack EO image understanding (EO-IU) capabilities, where spatial information dominates colour information (Matsuyama & Hwang, 1990). Such spatial features comprise topological (e.g. adjacency, inclusion) or non-topological (e.g. metric distance, angle measure), and even hierarchical ones (e.g. consists of, is part of) in agreement with the object-based image analysis (OBIA) paradigm (Blaschke et al., 2014). Existing EO-IU systems (EO-IUSs) fall short in transforming multi-source EO big data into comprehensive, timely and operational information products. For example, no EO data-derived Level 2 prototype product has ever been generated systematically at the ground segment (European Space Agency, 2015). By definition an EO Level 2 product consists of (a) an enhanced EO image corrected for atmospheric and topographic effects and (b) a general-purpose, user- and application-independent SCM, whose legend includes quality layers, such as cloud and cloud-shadow (European Space Agency, 2015).

### Scalable processing

SCBIR for big Earth data requires large-scale processing of images, while most existing approaches still handle the data as flat files. An alternative are array databases that can be queried by means of a declarative query language analogous to the Standard Query Language (SQL). Thus, the array database is able to perform internal optimizations, such as identifying the best access patterns using query plans, which can lead to a significant improvement in efficiency (Baumann & Holsten, 2011). In addition, input/output (IO) activities can be reduced by using a data model optimized regarding the data source, expected queries and the indexing of the database contents. Examples are time series analyses where potentially large amount of EO images have to be processed or when the specified AOI is smaller than one image or encompasses more than one image. In these query examples the handling of files using file-system and operating-system capabilities (such as locking) is inferior to the use of array databases, since it does not easily support typical concurrent large-scale processing properties such as query planning, load balancing, transactions or database indexing. Additional properties of databases can be exploited in an image querying application, for example, the technical and logical separation from the application logic, and, relevant for multi-user access, a better security through a finer granulated user rights management, a transaction manager which allows concurrent queries and database-inherent backup capabilities (Brinkhoff & Kresse, 2011). Although approaches for processing of remote sensing images in array databases or comparable systems which provide typical database capabilities are rare, some promising works have been carried out in recent years (Planthaber, Stonebraker, & Frew, 2012). Examples are the EarthServer (Baumann et al., 2016) and the Australian Geoscience Data Cube (AGDC, Purss et al., 2015). In the EarthServer, the array database implementation known as Rasdaman (Baumann et al., 2016) proved its capability to handle large-volume EO image bases.

In the remainder of this paper an innovative EO semantic querying (EO-SQ) system prototype, called *ImageQuerying* (IQ), is proposed as a proof of concept to work in series with an EO-IU subsystem, capable of multi-source EO big data spatiotemporal analytics as a pre-condition for SCBIR. The combined EO-SQ and EO-IU subsystems form an integrated EO image understanding and semantic querying (EO-IU&SQ) system. Within the EO-IU&SQ system design, the IQ system prototypical implementation in a distributed client-server architecture will be discussed in detail.

## EO-IU&SQ system design

**Figure 1.**
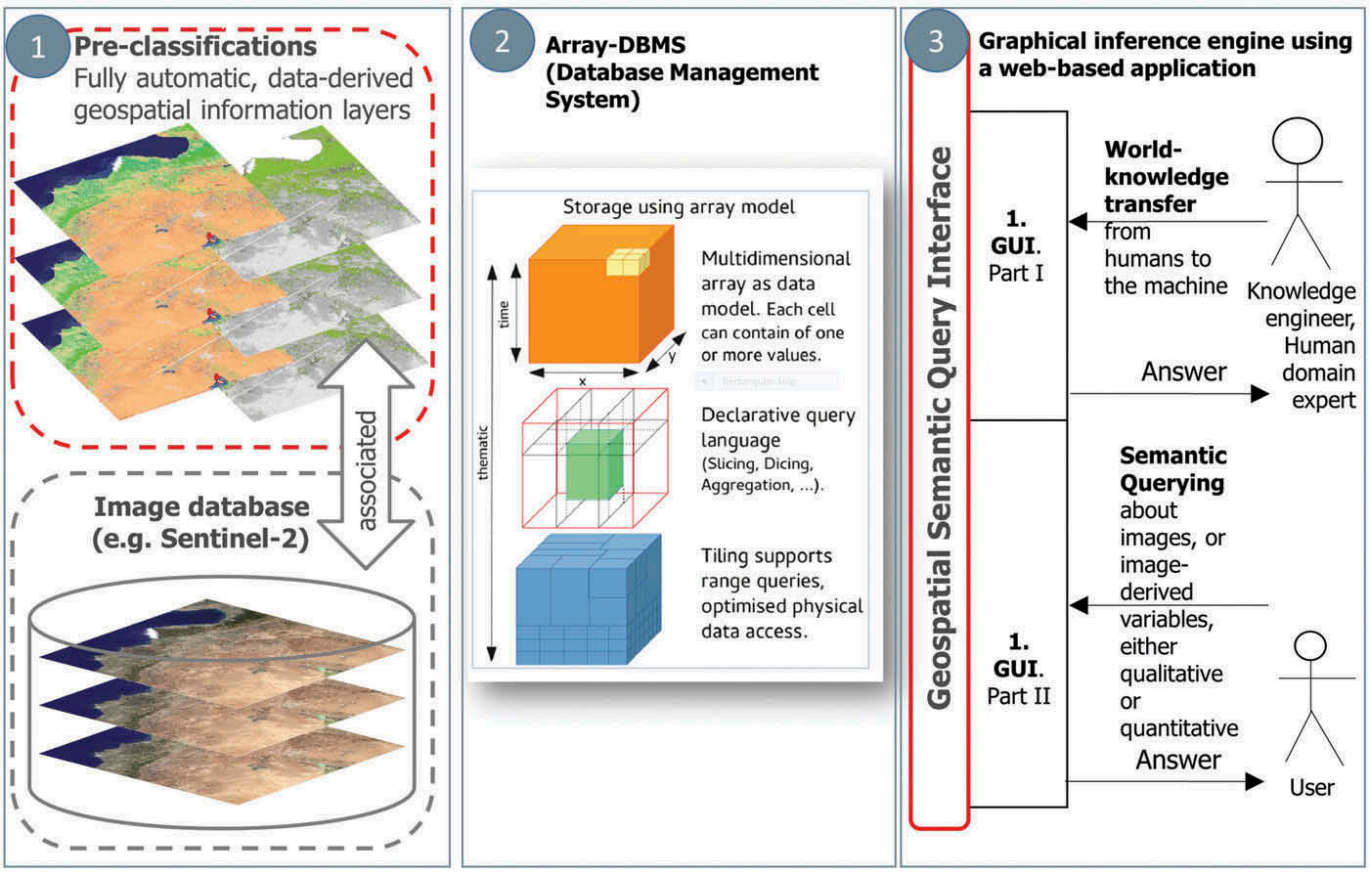
EO-IU&SQ system architecture. The EO-SQ subsystem is identified as sections (2) and (3).

**Figure 2.**
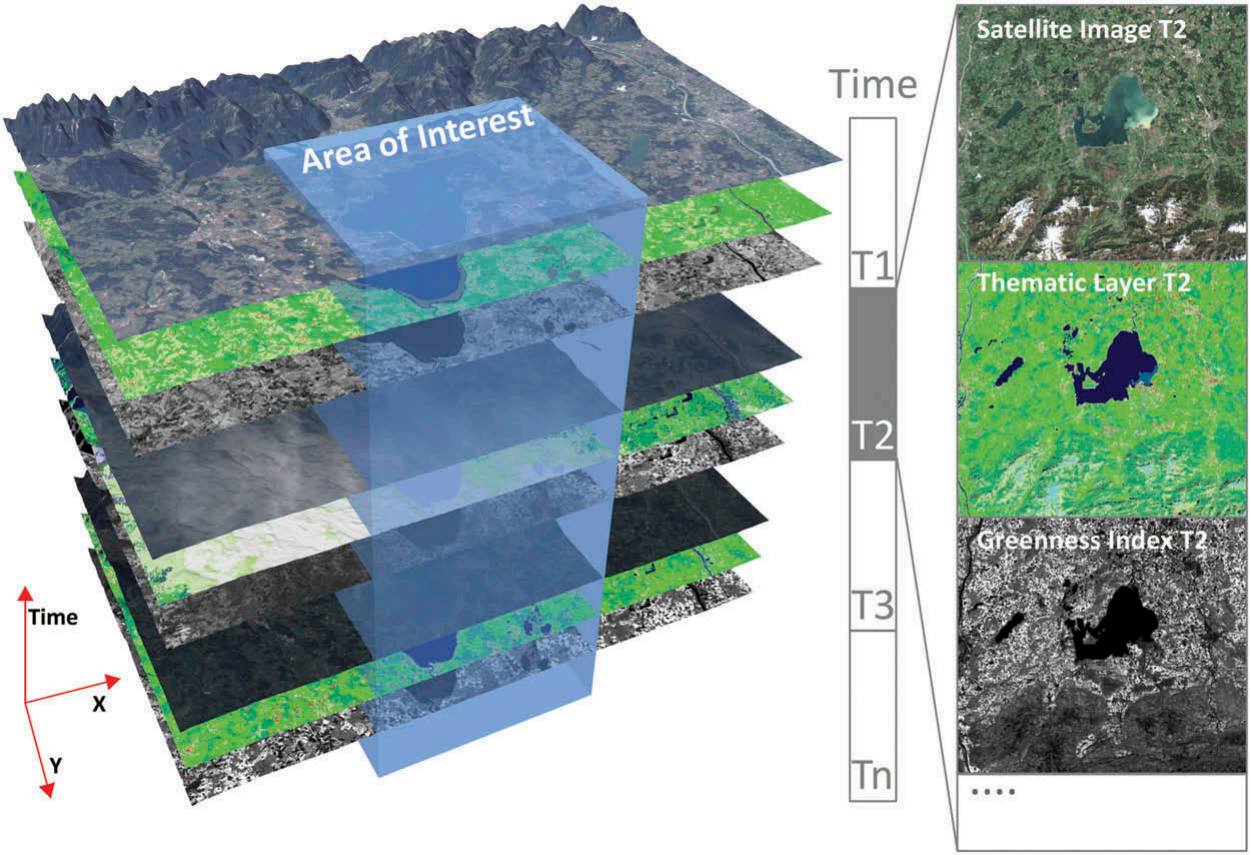
EO Level 2 information layers, either numeric/quantitative or categorical/qualitative, are automatically generated by the EO-IU subsystem and linked with the EO data to be employed as input by the EO-SQ subsystem for spatiotemporal semantic querying.

The integrated EO-IU&SQ system architecture (Baraldi, Tiede, Sudmanns, Belgiu, & Lang, 2016) consists of two subsystems: the EO-IU subsystem (section (1) in Figure 1) and the EO-SQ subsystem (sections (2) and (3) in Figure 1). These two subsystems share: (a) a fact base, where each EO image is stored together with its information products; (b) a knowledge base, encompassing physical laws, first-principle models, if-then decision rules, methods, processes, so on, eligible for generating new information from the fact base; and (c) an inference engine, which links the knowledge base to the fact base to infer new information. To be considered in operating mode the EO-IU subsystem requires EO data-derived information layers, to be generated automatically (without user interaction) and in near real time. Next, EO images are associated/linked with their information products (Figure 2), either nominal/categorical/qualitative, such as SCMs, or numeric/quantitative, such as spectral indexes, stored in a data cube model known as a space-time data cube within the array database (see also Figure 6). Finally, EO images and associated information products are input to the EO-SQ subsystem for spatiotemporal semantic querying.

**Figure 3.**
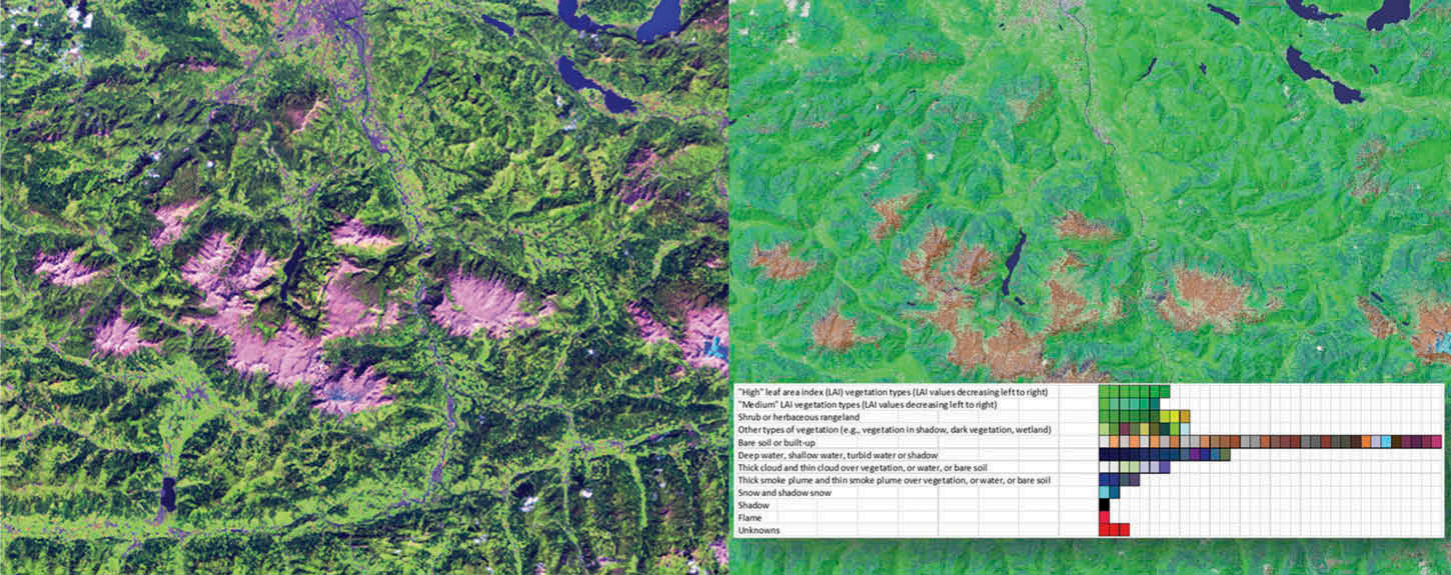
Left: Sentinel-2A (S2A) image of Salzburg, Austria, acquired on 13 August 2015, depicted in false colours: R = short wave infrared (SWIR), G = Near IR (NIR), B = Visible blue. No histogram stretching for visualization purposes. Right: Automatic SIAM mapping of the S2A image onto a legend of 96 MS colour names (spectral categories), depicted as pseudo colours (green as vegetation, blue as water or shadow, etc.).

**Figure 4.**
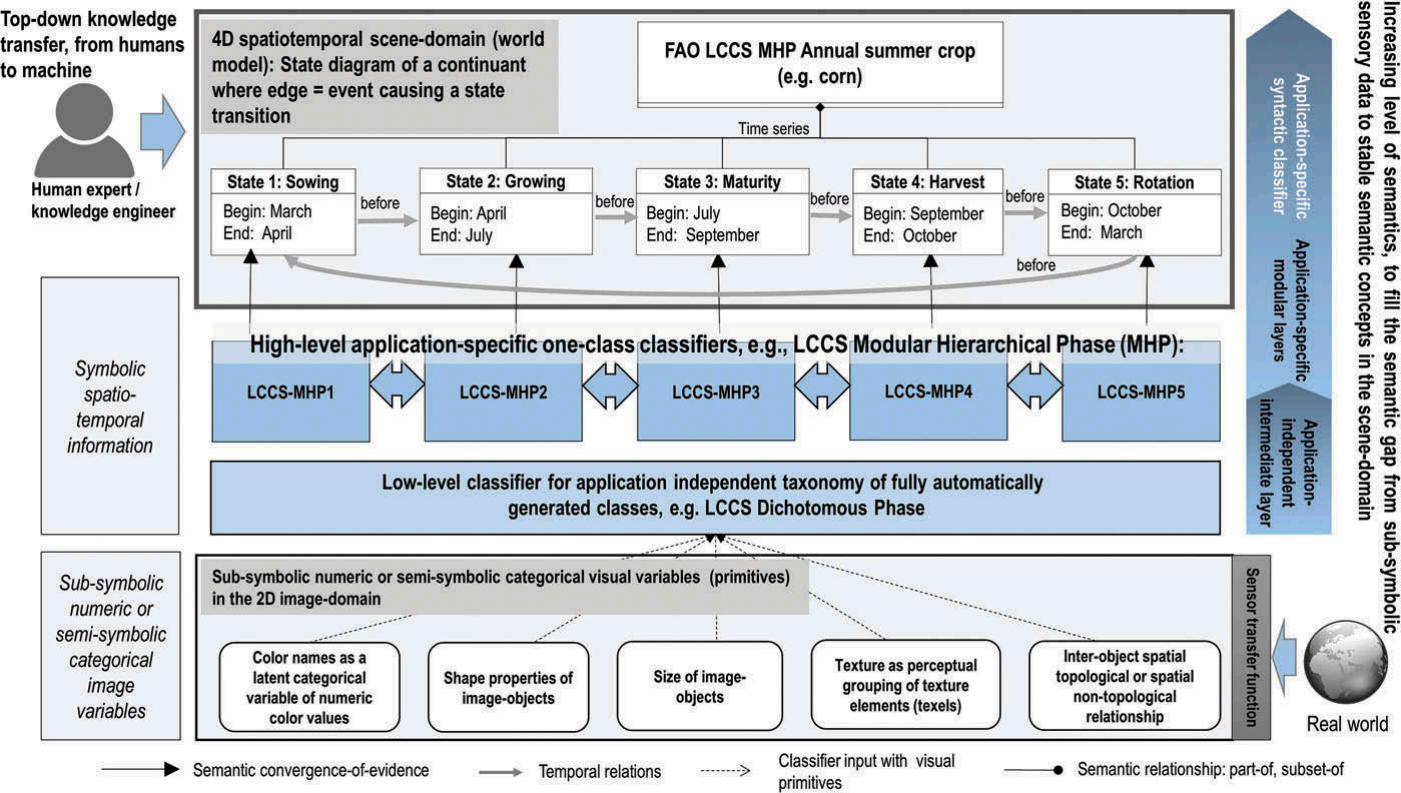
Semantic network of a real-world object with cyclic behaviour, specifically, a corn agricultural field in the northern hemisphere.

**Figure 5.**
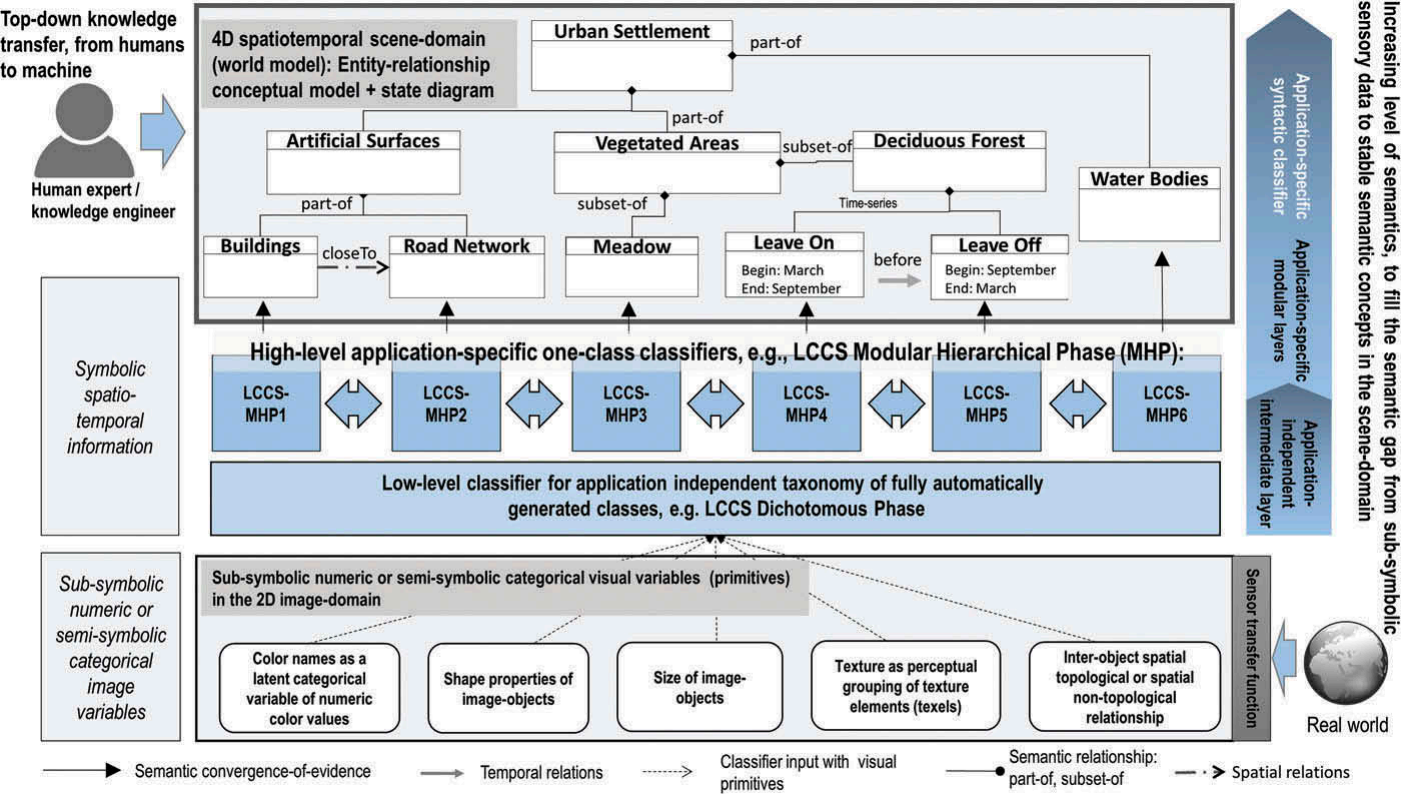
Semantic network of the aggregated object urban settlements based on two real-world persistent objects, specifically, artificial surfaces and water bodies and a periodic object vegetated areas, with the sub-object deciduous forest.

**Figure 6.**
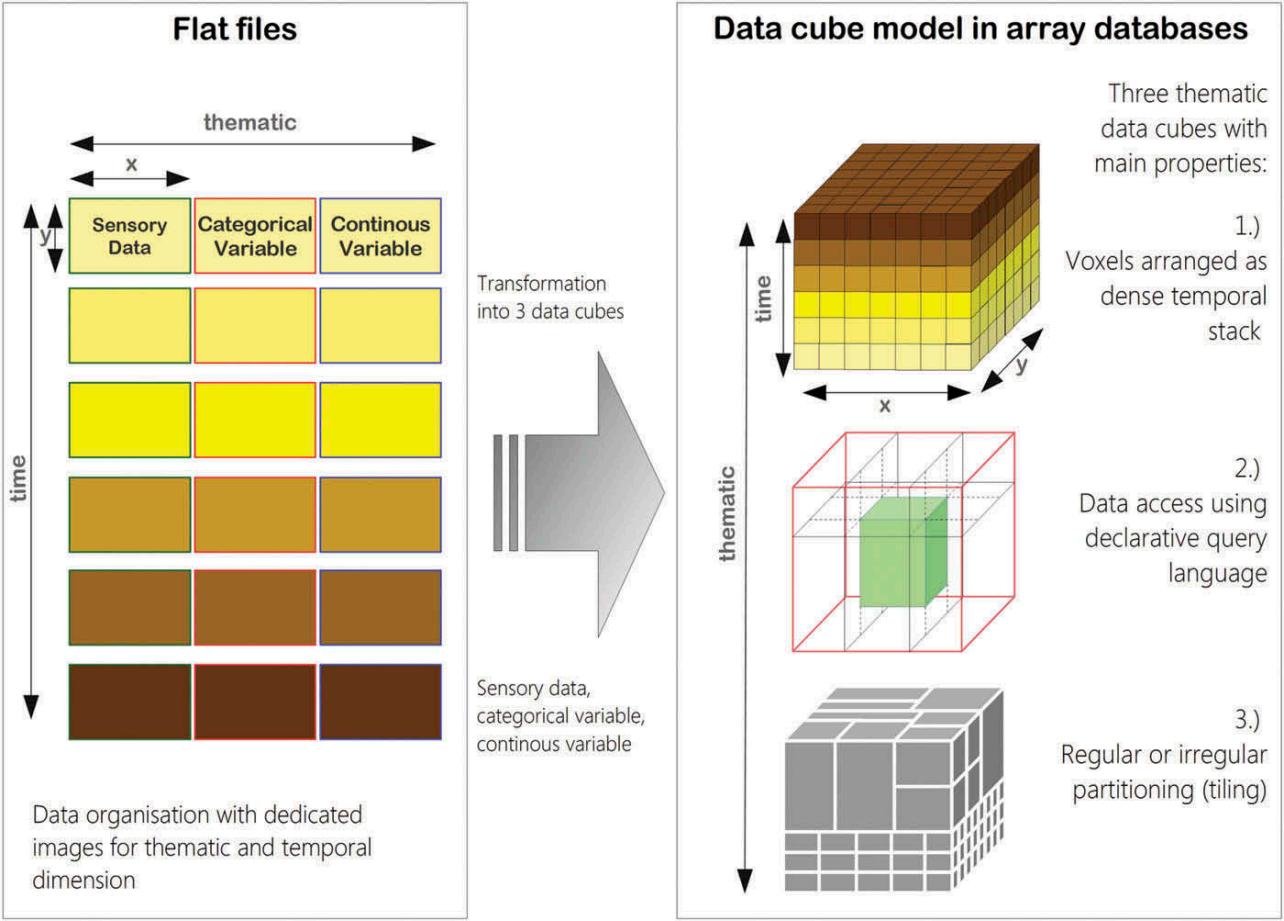
Storage using flat files versus storage of images in an array database.

### Generic rule base for low-level information layer generation

The generic rule base adopted by the EO-IU subsystem comprises a battery of automated EO data processing algorithms, requiring no user interaction to run in pipeline. They cope with EO data calibration, stratified (driven-by-knowledge) atmospheric and topographic effect removal (Baraldi, Gironda, & Simonetti, 2010), categorization of colour values into colour names for texel (superpixel) detection (Baraldi, Puzzolo, Blonda, Bruzzone, & Tarantino, 2006), wavelet-based image-contour detection, image segmentation (raw primal sketch) (Marr, 1982), texture segmentation (full primal sketch) (Marr, 1982), local shape analysis (Soares, Baraldi, & Jacobs, 2014) and stratified cloud/cloud-shadow detection (Baraldi, 2015). Some of these baseline algorithms, which were used in the prototypical implementation, are commented below.

#### EO image calibration, physical model-based colour naming and texel detection

Sensory data calibration (*Cal*) is a well-known “prerequisite for physical model-based analysis of airborne and satellite sensor measurements in the optical domain” (Schaepman-Strub, Schaepman, Painter, Dangel, & Martonchik, 2006). In practice, EO data *Cal* is mandatory to employ physical model-based and hybrid (combined deductive and inductive) EO-IUSs. To comply with the EO data *Cal* requirement, the proposed hybrid EO-IU subsystem employs a battery of sensor-specific EO image radiometric calibrators, for example, Landsat-5 to Landsat-8, MeteoSat, RapidEye, WorldView, etc., to transform digital numbers into physical units of measure, for example, top-of-atmosphere reflectance values.

Presented in the RS literature in recent years (Baraldi & Boschetti, 2012; Baraldi et al., 2010, 2006), the satellite image automatic mapper (SIAM) software product is a physical model-based decision tree (expert system) for deductive/top-down vector quantization (VQ) and VQ quality assessment in a multispectral (MS) reflectance space. VQ is a synonym of vector space polyhedralization (Cherkassky & Mulier, 1998). Since it is based on a priori knowledge available in addition to data, the SIAM expert system is fully automated, that is, it requires neither user-defined parameters nor training data to run. It maps each MS pixel onto one MS polyhedron, associated with an MS colour name in a predefined legend of MS colour labels. Per-pixel colour labels form a 2D multi-level VQ map in the (2D) image domain (Figure 3). This multi-level image is input to a well-posed (deterministic) two-pass connected component image labelling algorithm to extract a multi-level image segmentation map (Dillencourt, Samet, & Tamminen, 1992; Sonka, Hlavac, & Boyle, 1994). These image segments are connected sets of pixels featuring the same colour label that form texture elements (texels), whose detection occurs at the raw primal sketch of low-level vision (Marr, 1982). Recently, the computer vision community has used the term superpixel for perceptually meaningful units uniform in colour or brightness (Achanta et al., 2011).

#### Planar shape description

Given an image segmentation map consisting of planar objects, each planar object can be described in geometric (shape and size) terms by an mDMI set of planar shape indexes, such as area, characteristic scale, scale-invariant roundness, elongatedness, straightness of boundaries, simple connectivity and rectangularity (Soares et al., 2014). Shape descriptor values in the image domain are encoded as raster information layers in the array database to allow shape-related spatiotemporal queries, for example, change in size of a water body through time, distinguish similar classes based on form (see Use Case II below).

### Spatiotemporal conceptual modelling of real-world objects in the scene domain

In the 4D real world, observations (true facts) can be represented by *n*-tuples (space *x*, *y* and *z*, time *t*; “theme”; plus other numeric or categorical attributes, e.g. weight, size), where the 4-tuple (*x*, *y*, *z*, *t*) is the location in space and time of the observation, and the attribute “theme” identifies the real-world phenomenon or object being observed (Reis Ferreira, Camara, & Monteiro, 2014). “Theme” may account for semantics involved with the observed object or phenomenon. All possible combinations of attributes (space, time, theme) can be modelled according to the conceptualization of Reis Ferreira et al. (2014) as three data types, called *time series, coverage* and *trajectory*, where one attribute is measured, the second is fixed and the third is controlled. A *time series* represents the measured variations of a theme over a controlled time in a fixed location. A *trajectory* measures locations of a fixed theme over a controlled time. A *coverage* measures an attribute theme within a controlled spatial extent at a fixed time.

A conceptual world model is an ontology of real-world (geo-)spatiotemporal *objects/continuants* and *events/occurrence* derived from the three spatiotemporal data types time series, coverage and trajectory. In EO applications, a real-world object can be (a) a periodic object whose identity is fixed while its attributes change with a cyclic behaviour, that is, the object’s identity comprises a given sequence of different states in a fixed time periodicity, for example, a corn agricultural field whose growth cycle is decided by agricultural practices specific to a geographic region (Figure 4) or (b) persistent/non-periodic objects, for example, forested areas or lakes (Figure 5). An event is an individual episode with a definite beginning and an end (Belgiu, Sudmanns, Tiede, Baraldi, & Lang, 2016). It exists as a whole across the interval over which it occurs, either instant or durative. An event does not change over time. While an event can involve one or more objects, the same object can be involved in any number of events. Events can be (a) instant or (b) durative, including short-term transition events and slowly transient events. For example, in EO applications, LC-type *vegetated area* can change into any different LC type, such as bare soil, building or water, according to slowly transient events, such as urban sprawl due to changing policies or due to some short-term transition events, for example, wildfire or flooding. The world model can be graphically represented as a semantic network with LC classes as nodes and spatiotemporal relationships, including events, as links between nodes (Growe, 1999, cf. Figures 4 and 5).

A graphical user interface (GUI) allows users to easily select existing semantic queries/decision rules or to intuitively generate new ones. Each query instantiation is associated with an information pair, specifically, spatiotemporal scene-domain knowledge (e.g. target LC classes) and one set of sensor-specific transfer functions required to map scene-domain knowledge into image-domain knowledge. A query pipeline is considered a combination of spatial and temporal operators and/or standard algorithms whose inputs are qualitative/categorical information layers (e.g. SCMs) or quantitative/numeric variables (e.g. spectral indexes) available in the fact base. There are two types of semantic queries: (a) to accomplish SCBIR operations, where the fact base is investigated for EO image retrieval purposes, for example, retrieve EO images that are cloud-free across the selected geographic AOI and (b) to infer new information layers from the fact base, for example, detect flooded areas as a post-classification combination through time of available single-date SCMs.

### Array fact base

**Figure 7.**
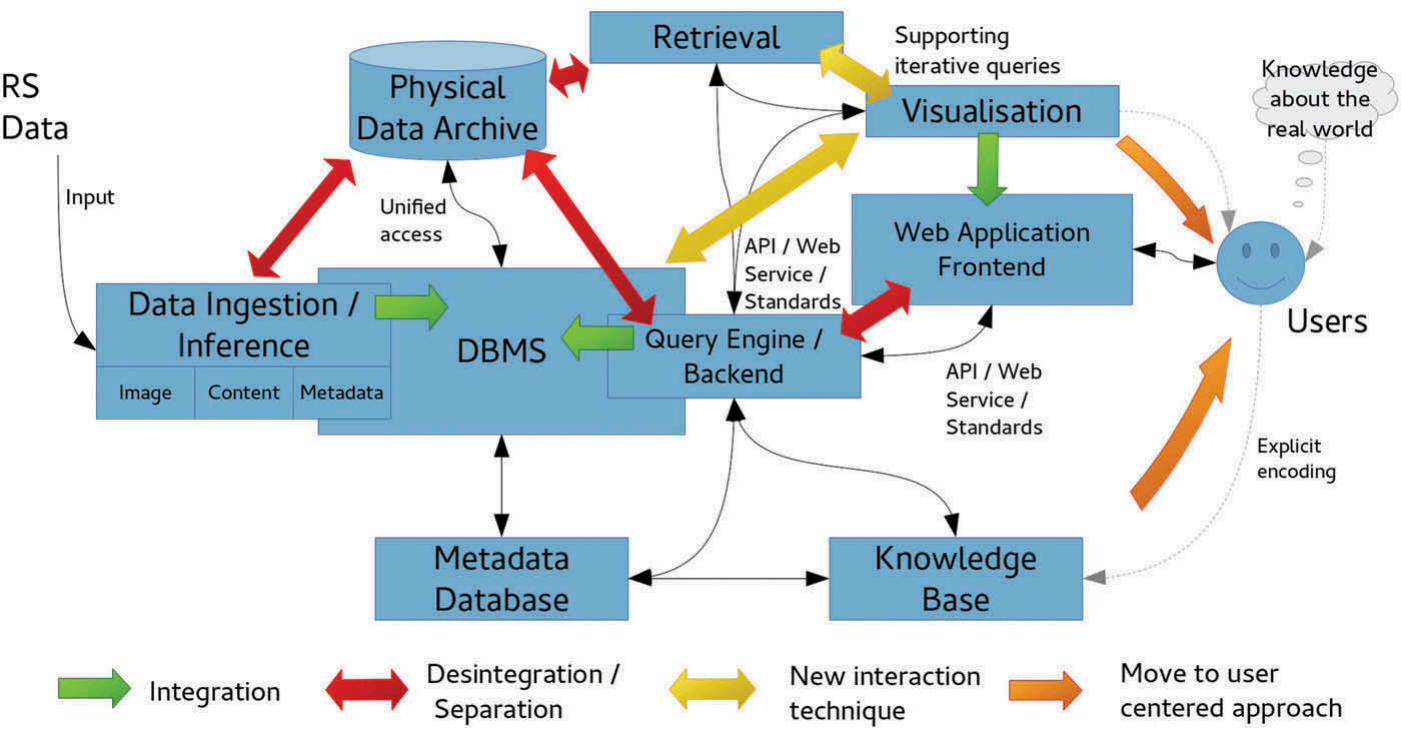
Client-server array database architecture.

To accomplish efficient geospatial data querying and analysis through space and time within a user-defined AOI and a target time interval, a fact base stores multi-sensor multi-temporal EO images, for example, Landsat-5/7/8 and Sentinel-2A time-series, together with their information products, either numeric, such as spectral indexes, or categorical, such as SCMs. This is realized within an array database (here: Rasdaman, Baumann et al., 2016), where multiple spatiotemporal data cubes are instantiated, which are in compliance with the Open Geospatial Consortium (OGC). All axes are described and accessible using coordinate reference systems to ensure inter-system harmonization and compatibility (Figure 6). In a spatiotemporal data cube the third dimension is time, defined in the Extensible Markup Language (XML) as a 1D temporal coordinate system using Unified Resource Identifiers (URIs). Time overlays the 2D spatial coordinate system, specified by European Petroleum Survey Group codes defined in XML using URIs. Similar to standard relational databases with its SQL, an array database consisting of data cubes can be queried by a declarative query language. In a database approach, storage-related characteristics, such as indexing, tiling and horizontal scaling as well as data models, can be investigated and optimized independently of the GUI. The data cube model has been proven to be scalable and reliable in operational applications (Baumann et al., 2016; Evans et al., 2015). These considerations make it best suited for the proposed EO-SQ system as storage backend. Semantic queries stored by the web-based query interface are accessible as OGC compliant web processing service (WPS) within the client-server architecture (Figure 7) and can be executed by any WPS client remotely on a single server or server cluster. By providing EO data processing capabilities together with “ready-to-analyse” data, this client-server architecture guarantees a fast-time response to queries.

## EO-SQ subsystem prototypical implementation

The proposed prototypical implementation of the EO-SQ subsystem, hereafter identified as IQ, is built upon the Rasdaman array database implementation. It stores every multi-source radiometrically calibrated EO image and its information layers as dense temporal stacks, known as space-time data cubes, whose third dimension is time. In greater detail, one EO image, its categorical and continuous information variables are stored in three different data cubes whose atomic element is a voxel. To improve IO performance, the data cube is automatically divided into smaller partitions (tiles) with the same dimensionality. Each partition is then stored as separate file on the hard disk. Additionally, large data cubes can be distributed on multiple servers. This distributed system can be scaled horizontally using the capability of the Rasdaman database to deploy multiple worker units coordinated by a management unit. Associated metadata, for example, for the spatial reference, are stored in an object-relational PostgreSQL database.

**Figure 8.**
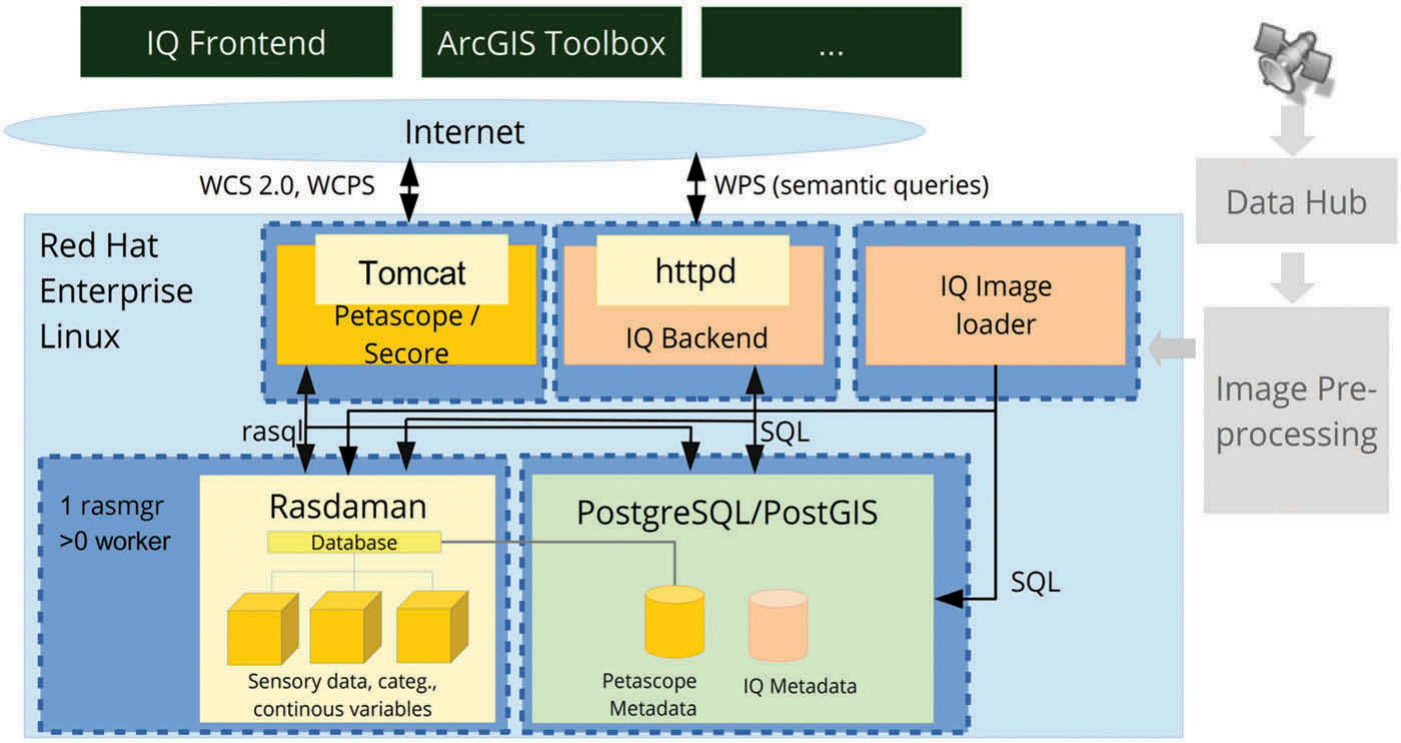
Web-based ImageQuerying (IQ) prototype. The term IQ is used for the prototypical implementation of the EO-SQ subsystem.

The main application tier is accessible over the internet using an Apache http webserver. It was written in python and developed in-house (Figure 8). The purpose of the main application tier is twofold. First, it translates queries formulated according to human reasoning and encoded as an XML by the IQ frontend into a valid database query to be executed against the database. Whenever an allowed user decides to store the query, the query is automatically published to other users in compliance with the OGC WPS. In this case the query can be executed with different AOIs and time spans by alternate clients, for example, ArcGIS, QGIS, as long as they provide an OGC compliant WPS client. This system is architecture- and software-agnostic, and it can be fully integrated into an existing workflow (Sudmanns, Tiede, Lang, & Baraldi, 2017a). Second, it provides API endpoints for system- and user management. The user management encompasses the user login and restrictions to accessible data sets as well as permissions to create and store queries. Registered users are the first of three user groups. Every query in the knowledge base is accessible by any registered user. Non-registered users are limited by a maximum AOI. Restriction to access is applied only to data sets and not to queries in order to foster the idea of the community- and knowledge-sharing-based approach. The second user group is expert users; they are a subset of the registered users having the permission to create and share queries. The third user group consists of administrators with permissions to manage the system as well as users. Administrators are allowed to create a new data set using an importer wizard within the IQ’s GUI. It allows to select the data source (e.g. Sentinel 2, Landsat), a geographical area (an AOI), a time span and available information layers. Next, system parameters are automatically fed into the IQ image loader which creates a single or routinely recurring instantiation of a process to download images from the official archive, transform them into products and store products into the fact base. Additionally, each data set can have a moderator, who is allowed to select single users or groups who have access to that data set.

EO big data require scalable and efficient processes to store, process and visualize EO images and products, but also affect quality assurance (QA). Besides automatic QA of sensory data, QA has to guarantee validity of a user’s semantic query/decision rule, whose input variables are images and products available in the fact base and whose functions are operators and processes available in the rule base. To enforce QA of decision rules, the EO-SQ subsystem delivers quality-ensuring metadata for each query, in agreement with the available 4D world model. Query-specific metadata are the possible target AOI, time interval and spatial, temporal, radiometric and spectral resolution of the imaging sensor, the name of the user who created the query and the time when the query was created. Query performance metrics are collected during each execution and updated regularly.

### Use case examples

The IQ’s GUI allows a user (1) to select the AOI and (2) the target time period and (3) to create or select via graphical elements the decision-rule pipeline for semantic querying the fact base. Once a decision rule is executed, the query results are shown in the image domain and/or as summary statistics. The following sections discuss four examples of semantic spatiotemporal queries instantiated by users through the IQ’s GUI.

#### Use case I – LC change detection through time

**Figure 9.**
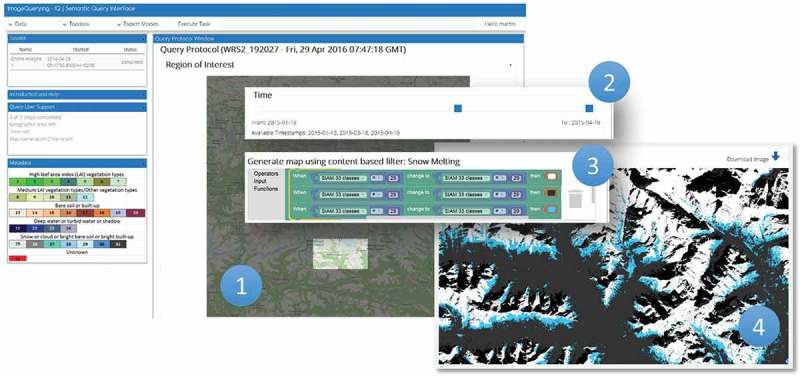
Semantic querying to infer new information layers from the fact base, here: snow cover analysis. For a detailed description see main text.

A snow cover analysis through time is conducted across an alpine area, specifically, the Hohe Tauern National Park in Austria (Figure 9, point 1). The spatiotemporal semantic query (Figure 9, points 2 and 3) shows the selected time span as well as the user-defined LC change (LCC) classes to be extracted from the fact base. Such a query can be easily saved, modified and shared with other users. The GUI shows the query output product, which can be downloaded as a geoTiff or re-used for further queries. The output map areas depicted in blue/white identify loss/persistent snow cover detected from January to April 2015 (Figure 9, point 4).

#### Use case II – planar shape descriptors to infer high-level LC classes from Level 2 products

**Figure 10.**
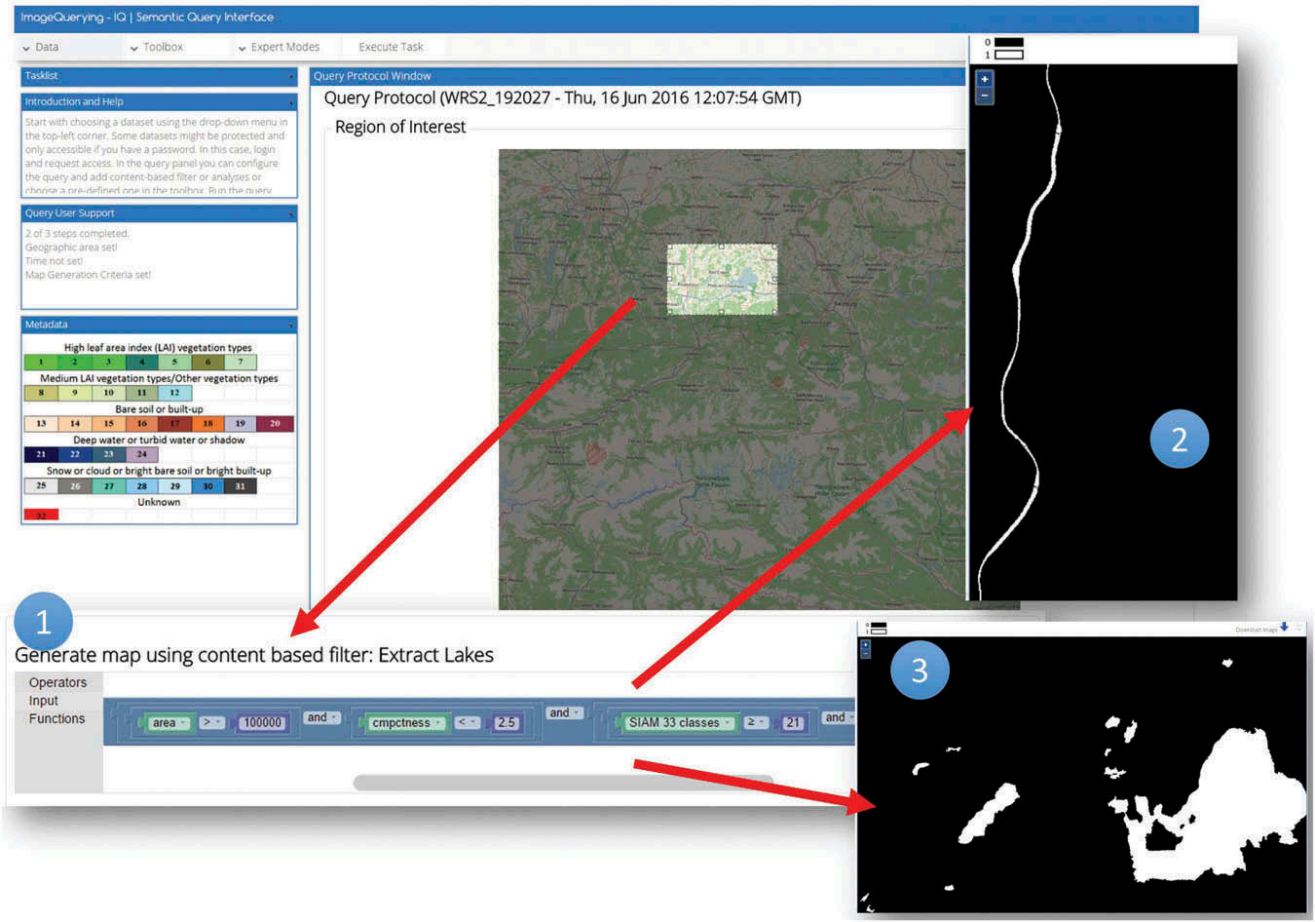
Semantic querying to infer new information layers from the fact base. A Landsat-8 image time series is analysed by a semantic query to distinguish between lakes and rivers as water areas (spectral information) of a target size and compact/elongated form (planar shape descriptors) from the database of EO-derived information layers.

In the hierarchical two-phase Food and Agriculture Organization of the United Nations (FAO) – Land Cover Classification System (LCCS) (Di Gregorio & Jansen, 2000), an application-independent general-purpose eight-class LCCS-dichotomous phase (DP) taxonomy is preliminary to the LCCS modular hierarchical phase (MHP), consisting of a hierarchical battery of application- and user-specific one-class LC classifiers (see also Figures 4 and 5 of the semantic network description). In agreement with a hierarchical LCCS taxonomy, high-level LC classes Lake and River were extracted from Level 2 SCMs, by incorporating shape descriptors in a user’s query. More specifically, LC lake and river candidate areas were selected as water objects in the image domain whose area in pixel units was within a given physical model-based range of values and whose shape compactness in the user query (Figure 10 point 1) is fuzzy *high* (lake, Figure 10 point 3) or “low” (river, Figure 10 point 2), elongatedness is *low* and *high* respectively, so on. The output lake/river-from-water LC binary mask is a geoTiff file, to be stored or reused in further queries (Figure 10).

#### Use case III – cloud-free SCBIR

In contrast to the existing EO data portals, this use case shows, how, within a user-defined AOI and time interval, all multi-source EO images available in the fact base can be retrieved, where no cloud (or a specific percentage) is found across the AOI, based on the available Level 2 SCMs. In the implemented IQ prototype, image retrieval queries turn back the matching image IDs and associated coverages which are accessible using the OGC Web Coverage Service (WCS). Other examples for such an AOI-based SCBIR are searching for images showing flooded areas/burnt areas/deforested areas or similar in the selected AOI.

#### Use case IV – flood extent mapping and aggregation through time

**Figure 11.**
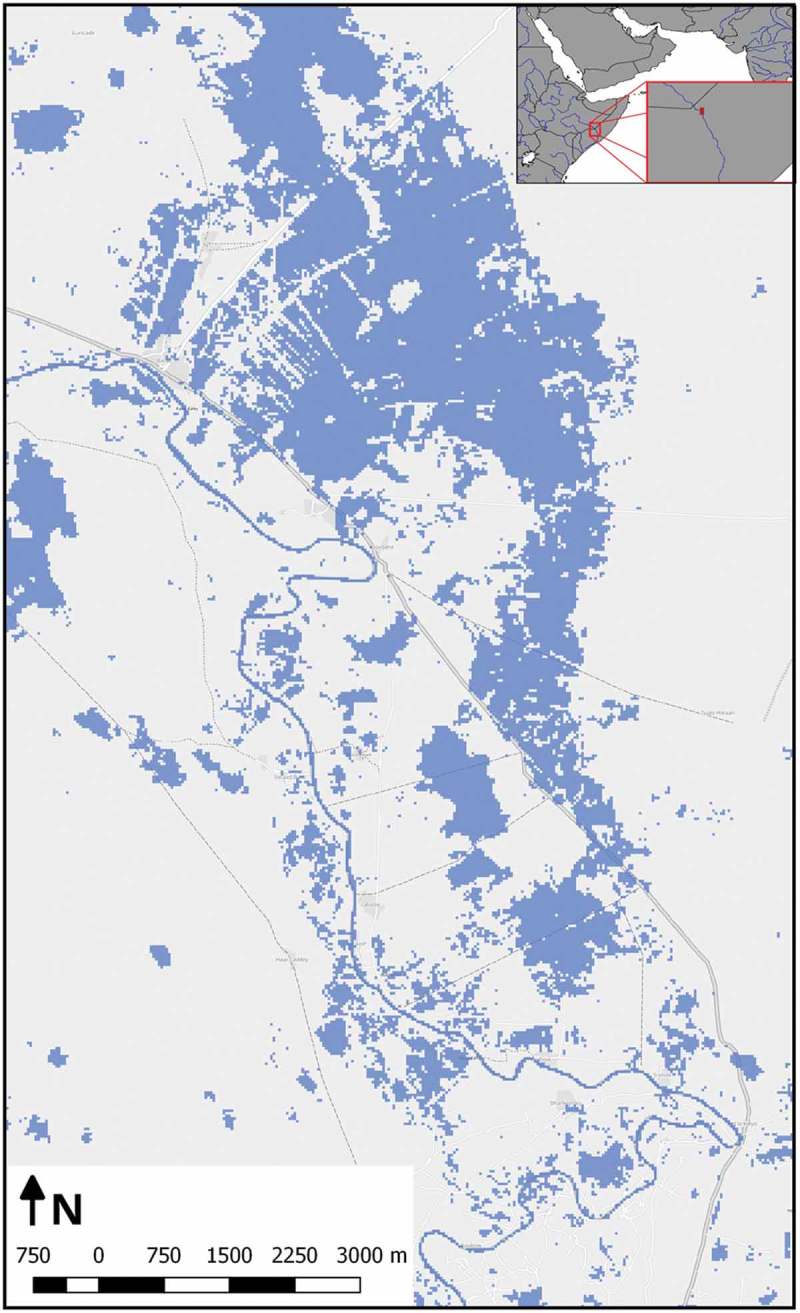
Extracted flood mask in a study area in Somalia using a long time series of optical EO images. Background basemap is copyrighted by OpenStreetMap contributors and available from https://www.openstreetmap.org.

For a test area in Somalia, all 78 available Landsat 8 scenes between 2013 and September 2016 were pre-processed and fed into the database allowing queries to detect flood events and their recorded extent as well as a spatial aggregation of flooded areas as an indicator for flood-prone areas through time (Sudmanns, Tiede, Wendt, & Baraldi, 2017b). Figure 11 shows the result of such a query, aggregating areas covered by water at least once in all Landsat 8 images from January 2016 until September 2016. The use case illustrates the scalability of the approach, documented by the following performance indicators: The initial pre-processing and the ingestion into the database required around 5–10 min per Landsat image on a standard PC, which is in the range of the usual download time for an image of this size, can be linearly reduced by parallelization. For a subset of an area of approximately 7 × 9 km with a pixel size of 30 m, more than five million of observations in space and time needed to be accessed and processed within the ad hoc query to conduct the analysis. In this case, the analysis took less than 10 s for the database query through time. A single stand-alone node of the Rasdaman array-database management system (DBMS), operated on a virtual machine running Red Hat Enterprise Linux) was used with four virtual CPUs (2.50 GHz clocking) and 16-GB virtual RAM. No dedicated high-performance storage system was utilized for this feasibility study.

## Conclusions

This paper started from the statement that existing EO CBIR systems lack EO-IU capabilities and therefore fail to support semantic querying. To our best knowledge, no EO SCBIR system in operating mode has ever been developed by the RS community. A prototypical implementation of an EO-SQ subsystem (identified as IQ) plugged-in an innovative EO-IU&SQ system was proposed as a proof of concept. Capable of providing every EO image stored in the database with EO Level 2 products generated automatically (without user interaction) and in near real time, the EO-IU subsystem is preliminary to IQ. Within the proposed EO-IU&SQ system architecture, the EO-IU and EO-SQ subsystems, their algorithms and their implementations can be modified or replaced to accomplish SCBIR capabilities in operating mode.

Further research and development will be focused on six areas: (1) increase the 4D spatiotemporal world model; (2) develop and implement an algebra to describe spatiotemporal data types and operations in a language-independent and formal way inspired to Reis Ferreira et al. (2014); (3) augment the GUI for semantic query selection and writing; (4) improve efficiency of on-the-fly EO image processing capabilities within array databases, for example, to compute shape descriptors of image–objects selected by spatiotemporal queries and improve querying spatial and temporal relations of the objects; (5) validating the outcome and process QIs in a multi-scale EO big data scenario; (6) improving outcome and process QIs in the knowledge base, with special emphasis on (a) cloud/cloud-shadow quality layer detection, (b) EO Level 2 product generation, where the general-purpose application- and user-independent Level 2 SCM’s legend coincides with the eight-class LCCS-DP taxonomy (Di Gregorio & Jansen, 2000).
